# Using a virtual flipped classroom model to promote critical thinking in online graduate courses in the United States: a case presentation

**DOI:** 10.3352/jeehp.2022.19.5

**Published:** 2022-02-28

**Authors:** Jennifer Tomesko, Deborah Cohen, Jennifer Bridenbaugh

**Affiliations:** Department of Clinical and Preventive Nutrition Sciences, School of Health Professions, Rutgers The State University of New Jersey, Newark, NJ, USA; Hallym University, Korea

**Keywords:** Distance education, Feedback, Health occupations, Problem-based learning, Thinking

## Abstract

Flipped classroom models encourage student autonomy and reverse the order of traditional classroom content such as lectures and assignments. Virtual learning environments are ideal for executing flipped classroom models to improve critical thinking skills. This paper provides health professions faculty with guidance on developing a virtual flipped classroom in online graduate nutrition courses between September 2021 and January 2022 at the School of Health Professions, Rutgers The State University of New Jersey. Examples of pre-class, live virtual face-to-face, and post-class activities are provided. Active learning, immediate feedback, and enhanced student engagement in a flipped classroom may result in a more thorough synthesis of information, resulting in increased critical thinking skills. This article describes how a flipped classroom model design in graduate online courses that incorporate virtual face-to-face class sessions in a virtual learning environment can be utilized to promote critical thinking skills. Health professions faculty who teach online can apply the examples discussed to their online courses.

## Introduction

### Background/rationale

In traditional classrooms, students listen to lectures and accomplish most assignments outside of the classroom. Flipped classrooms reverse traditional course components such as lectures, projects, and assignments. This pedagogy highlights pre-recorded lectures as the primary mechanism for teaching, which is often complemented with additional videos, podcasts, and readings housed in a virtual learning environment. During face-to-face class time, students have the opportunity to ask questions and apply their knowledge through active learning techniques, including but not limited to discussion of case studies, role-playing, or problem-based learning. Autonomy is encouraged, as students are active participants in their learning. Unlike teaching methods in traditional classrooms, which are more passive, the expectation is that students come to the flipped classrooms prepared; therefore, a greater responsibility lies with the students to understand the material. Course faculty facilitate face-to-face class activities, provide feedback in real-time, and engage students while assisting in their critical thinking skills development. Flipped classrooms may improve students’ ability to synthesize complex information and increase their critical thinking skills [1].

A meta-analysis by Hew and Lo [2] examined a flipped classroom approach compared to traditional teaching methods in health professions students and found significant improvements in student learning performance. Similarly, in a meta-analysis conducted by Shi et al. [3], researchers found improved cognitive learning in college students educated with a flipped classroom model in medical, science, and social science courses. Birgili et al. [4] explored trends in flipped classrooms and found positive impacts on students’ cognitive thinking skills in higher education students. Additionally, flipped classroom models have increased critical thinking skills in nursing students [5,6].

### Objectives

This study aims to provide guidance to health professions faculty with examples of how to use flipped classrooms that incorporate virtual face-to-face class sessions in a virtual learning environment to promote critical thinking skills. A discussion follows on the virtual flipped classroom model design and its use in an online graduate nutrition curriculum. This guidance was developed between September 2021 and January 2022 at the School of Health Professions, Rutgers The State University of New Jersey.

## Establishing a virtual flipped classroom

**Fig. 1.**
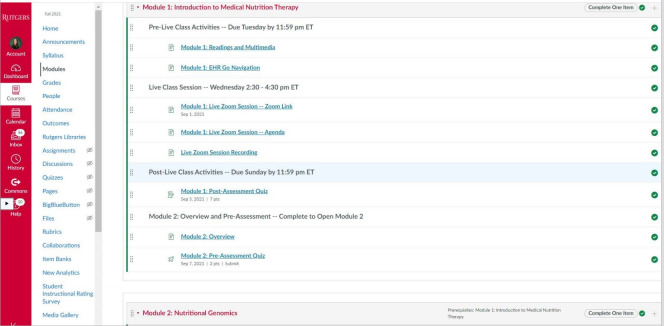
Learning management system screen view for a weekly module in an online course of medical nutrition therapy between September 2021 and December 2021 at the School of Health Professions, Rutgers The State University of New Jersey.

There are key components to ensure that flipped classrooms are established to promote critical thinking skills. Virtual face-to-face learning activities should incorporate problem-solving and reflective practice via weekly synchronous virtual face-to-face class sessions using a web conferencing program such as Zoom. In addition, course learning objectives should address higher-level thinking and communication skills, critical thinking skills, and teamwork. Faculty must educate students on the objectives of the flipped classroom model before initiation of their courses to provide them with a clear understanding of faculty expectations and ensure accountability. It is necessary to consult the institution’s instructional designers for assistance in the initial design of each course to establish a standardized course structure and accessibility. Furthermore, consideration should be given to providing a designated area for pre-class, face-to-face, and post-class activities in each weekly course module (Fig. 1) to promote organization and student engagement. Students who are satisfied and content in a learning environment may have better academic outcomes. Table 1 describes examples of learning activities from courses in a graduate nutrition curriculum.

## Pre-class activities for a virtual flipped classroom

Faculty will provide pre-class material or foundational knowledge content to students through pre-recorded lectures that students can access through their virtual learning environment. This content should be available to students before their virtual face-to-face class session; adequate pre-class time is necessary to complete these learning activities. Dividing video lecture content into concise material is more manageable, with a maximum time of 15 minutes per recording. Students view shorter videos more often and are more likely to watch their entirety, resulting in more excellent material retention than longer videos [7]. It is essential that all pre-recorded videos can be easily downloaded from the virtual learning environment and have user control functions to pause, play, or rewind the videos. Many students prefer to access learning materials on their own time when it is most conducive to their schedules. Additional content may include readings from textbooks, journal articles, website links, YouTube videos, or other interactive web-based tutorials to complement pre-recorded lectures. Students are responsible for learning foundational knowledge, which helps facilitate higher levels of engagement and critical thinking skills during the virtual face-to-face class sessions. The following example of a pre-class activity can be adapted to any health professions curriculum.

Example: In an online medical nutrition therapy course, students take a 5-question ungraded quiz before class, which unlocks weekly module content (Supplement 1). The quiz serves as a baseline knowledge assessment of the weekly content to assist students in focusing their learning. Students have approximately 3 hours of foundational content to review prior to the virtual face-to-face class session.

## Face-to-face class activities for a virtual flipped classroom

**Fig. 2.**
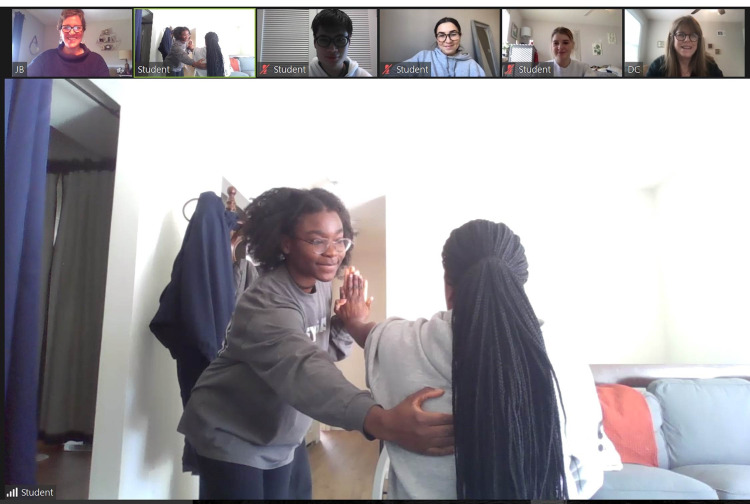
Student performing a fat and muscle examination during a virtual face-to-face class with faculty providing real-time feedback and direction in an online course on medical nutrition therapy between September 2021 and December 2021 at the School of Health Professions, Rutgers The State University of New Jersey.

The virtual face-to-face class sessions create a bridge between the pre-class lecture material and practical applications that resemble simulated clinical scenarios and can assist in developing critical thinking skills. This time reinforces foundational knowledge and key concepts through the application of active learning and problem-solving. These activities align with the objectives communicated in the pre-class content. Active learning in the classroom includes simulations (Fig. 2), role-playing, case studies, small group discussion, and live presentations. The virtual flipped classroom approach creates a student-centered structured class environment. The following examples of in-class activities can be adapted for use in any health professions curriculum (Fig. 2).

Example 1: Students are divided into pairs in breakout rooms during the virtual face-to-face class session during an online nutrition assessment and physical examination course and practice food and nutrition-related history-taking techniques. Once back in the large group, students share their reflections on the experience and discuss how to improve their techniques in preparation for supervised practice. Students reflect or debrief and faculty guide them through their experience to understand, analyze, and synthesize what they learned (Supplement 6).

Example 2: In an online ethics, professionalism, and leadership course, virtual face-to-face class activities include large- and small-group discussions that apply concepts from pre-class readings, debates on ethical issues in the clinical setting and student presentations related to current nutrition controversies. Debates allow students to argue about controversial problems using higher-order thinking skills. Both the actual debate and the preparation for the debate are considered active learning tools and can assist students in critical thinking skills development [8].

Example 3: An online course on ethically culturally sensitive interviewing and counseling integrates various role-playing encounters in the virtual face-to-face class session. The course instructors visit each breakout room to provide immediate feedback and redirection if necessary. Role-playing scenarios that involve a patient and health practitioner increase critical thinking skills [9].

Example 4: Students develop skills in formulating research questions in an online evidence-informed practice and scientific inquiry course. Students are placed in breakout rooms in pairs during the virtual face-to-face class sessions, receive a short clinical scenario, and work as a team to develop a clinical research question. Upon return to the main room, critical thinking skills are exhibited as student pairs present their case, research question, rationale, and classmates critique each other’s research question. Incorporating a flipped classroom model with pre-recorded evidence-based practice lectures and small group discussions as an active learning strategy during classroom sessions may promote critical thinking skills [10].

## Post-class assignments and assessment approaches

Assessment of learning and critical thinking skills can be achieved with formative and summative assessments using active learning techniques, quizzes, exams, self-assessment, case study presentations, and assignments that emphasize analysis, synthesis, and evaluation of student performance. Formative assessments are designed to provide the learner with quick and informative feedback, while summative assessments are utilized to evaluate knowledge and comprehension. Assessment of student performance occurs in virtual face-to-face class sessions and post-class activities. Active learning techniques such as simulation, role-playing, live presentations, and self-assessment of physical examination all occur during virtual face-to-face class sessions. It allows the faculty to provide the student with quick and informative feedback in real-time, fostering effective learning. After the virtual face-to-face class sessions, students complete post-class assignments to enhance critical thinking skills, allowing faculty to assess student performance further. Post-class formative and summative assessment strategies in flipped classrooms include short post-quizzes where students obtain immediate feedback and the rationale for the answers. Additional examples that can be assigned in many health professions programs include case studies that incorporate documentation of patient care plans, formulating research questions, and recording physical examinations performed on a volunteer. Each assignment is graded and returned to the student with extensive narrative feedback, which allows the student to improve foundational knowledge and critical thinking skills.

## Conclusion

The flipped classroom model has been found to promote critical thinking skills in health professions students [1,5,6]. Health professions faculty can mentor students in synthesizing and analyzing foundational material, which students have reviewed in preparation for face-to-face class sessions. The flipped classroom model also allows for more “hands-on” activities and simulations during face-to-face class sessions that emphasize active learning techniques, improving understanding and enhancing the application of the material. The virtual flipped classroom model may benefit health professions faculty who teach online. This model can be modified to complement any subject matter and may facilitate the development and application of critical thinking skills.

## Figures and Tables

**Table 1.** Learning activities to promote critical thinking for a virtual flipped classroom in a nutrition curriculum

Example: online course module	Student: pre-class activities	Virtual face-to-face class: activities	Student: post-class activities^a)^
Clinical and Medical Nutrition Therapy I:EN Module	EN pre-quiz; EN chapter and article readings; 4 (15–20 min) recorded lectures on EN indications, initiation, prescription and monitoring	EN large group discussion and sample EN calculations; EN case scenarios in Zoom breakout rooms; small group presentations and discussion	EN post-quiz; (Supplement 1) EN calculation assignment
Pharmacology Seminar: Drugs for CNS Module	CNS pre-quiz; CNS guidelines and article readings; 3 (20 min) recorded lectures on drug therapy; 1 (15 min) podcast on CNS drug side effects	Zoom breakout rooms to collect NCP assessment data from simulated EHRs for a CNS case; students return to the main Zoom room for NCP presentations and group case discussions	CNS post-quiz (Supplement 2); CNS NCP clinical case study
Evidence Informed Practice and Scientific Inquiry: Critically Appraising the Literature Module	Chapter readings and website tutorial on critical appraisal; 1 (20 min) recorded lecture on critical appraisal; assigned journal article reading for live session activity	Critical appraisal large group discussion; Zoom breakout rooms to critically appraise their assigned journal article; students return to the main Zoom room as faculty facilitate the critical appraisal review	Critical appraisal (Supplement 3) of 2 journal articles for an evidence analysis project assignment
Nutrition Assessment and Physical Examination: NFPE Module	NFPE pre-quiz; NFPE article readings; recorded lectures: on malnutrition screening, fat and muscle orofacial and cranial nerve assessment; 1 (10 min) video on conducting NFPE exam	Zoom breakout rooms to complete an NFPE on a volunteer (Supplement 4); faculty and student peers observe the NFPE encounter; faculty evaluate the student and record on a scoring rubric; debriefing in the main Zoom room	Student self-evaluation and reflection of NFPE video
Ethically and Culturally Sensitive Interviewing and Counseling: Patient Centered Counseling Individual Module	MI chapter and article readings; MI online simulation practice; 4 (3–5 min) videos on MI technique (Supplement 5)	Zoom breakout rooms in groups of 3; students rotate and role-play as a counselor, patient and observer; faculty observe the encounter; debriefing in the main Zoom room	Students write a self-reflection in a learning diary

EN, enteral nutrition; CNS, central nervous system; NCP, nutrition care process; EHR, electronic health record; NFPE, nutrition-focused physical examination; MI, motivational interviewing.

a) All post-class activities are graded.

## Supplementary materials

Supplementary files are available from Harvard Dataverse: https://doi.org/10.7910/DVN/ER5K8K.

Supplement 1. A 5-question ungraded pre-quiz before and a 7-question graded post-quiz after class in an online medical nutrition therapy course between September 2021 and December 2021 at the School of Health Professions, Rutgers The State University of New Jersey.

Supplement 2. A 5-question ungraded pre-quiz before and a 7-question graded post-quiz after class in an online pharmacology seminar course between September 2021 and December 2021 at the School of Health Professions, Rutgers The State University of New Jersey.

Supplement 3. The Academy of Nutrition and Dietetic’s Evidence Analysis Library critical appraisal form used to critically appraise research articles between September 2021 and December 2021 at the School of Health Professions, Rutgers The State University of New Jersey.

Supplement 4. Video of nutrition-focused physical examination module from Table 1 in a Zoom breakout room available from: https://youtu.be/DqgfazuwurQ.

Supplement 5. Videos used to teach motivational interviewing in an online course on ethically and culturally sensitive interviewing and counseling between September 2021 and December 2021 at the School of Health Professions, Rutgers The State University of New Jersey. https://simulations.kognito.com/changetalk/.

Supplement 6. Example video of food and nutrition-related history in a Zoom breakout room https://youtu.be/BuhiVtndl-8.

Supplement 7. Audio recording of the abstract.
