# Supplementary material for: Using a virtual flipped classroom model to promote critical thinking in online graduate courses in the United States: a case presentation
Source: J Educ Eval Health Prof. 2022 Feb 28;19:5. doi: 10.3352/jeehp.2022.19.5 (PMC9008223; doi:10.3352/jeehp.2022.19.5)
Supplement: Supplementary file 1 — Supplement 1. A 5-question ungraded pre-quiz before and a 7-question graded post-quiz after class in an online medical nutrition therapy course between September 2021 and December 2021 at the School of Health Professions, Rutgers The State University of New Jersey. [file jeehp-19-05-suppl1.pdf]

NUTR 5361 Clinical Nutrition and Medical Nutrition Therapy I Week 9 Pre-Assessment

# of Questions: 5
Total Exam Points: 5.00

Question #: 1

You receive a consult for a patient that presents with hypokalemia and new episodes of diarrhea. The patient began taking a Potassium Elixir when the diarrhea started. The patient has been receiving a tube feeding without any intolerance issues for the past 6 months. What do you recommend?

Item Weight: 1.0

Question #: 2

You receive a consult from a patient newly admitted to the hospital from a nursing home. The patient has had a poor oral intake for weeks, and a significant weight loss of 10% of their normal body weight in the past month. Their biochemical assessment reveals that the Magnesium, Phosphorus, and Potassium are critically low, and the blood glucose is elevated. What syndrome may this patient be at risk of?

Item Weight: 1.0

Question #: 3

You receive a consult for a patient in the ICU for a tube feeding. They are intubated and receiving mechanical ventilation after a motor vehicle accident. They are in critical condition, and have an advance directive that states they wish for artificial fluids and hydration. What feeding route do you recommend as your intervention?

Item Weight: 1.0

Question #: 4

You have assessed a patient in the hospital setting that has not been eating well. The patient's food and nutrition related history reveals an intake of <25% of their estimated nutrient requirements for about a month prior to hospitalization. You determine that this patient is a candidate for nutrition support. Which type of nutrition support will you recommend as a first intervention?

Item Weight: 1.0

Question #: 5

You have a patient that has hyperglycemia. They have no past medical history of Diabetes, but they have recently been hospitalized for pneumonia. They are intubated, and you are consulted to start a tube feeding. What type of tube feeding do you recommend?

Item Weight: 1.0

NUTR 5361 Clinical Nutrition and Medical Nutrition Therapy I Week 9 Post-Assessment

# of Questions: 7

Total Exam Points: 7.00

Question #: 1

You receive a consult for a patient that presents with hypokalemia and new episodes of diarrhea. The patient began taking a Potassium Elixir when the diarrhea started. The patient has been receiving a tube feeding without any intolerance issues for the past 6 months. What do you recommend?

Item Weight: 1.0

Question #: 2

You receive a consult from a patient newly admitted to the hospital from a nursing home. The patient has had a poor oral intake for weeks, and a significant weight loss of 10% of their normal body weight in the past month. Their biochemical assessment reveals that the Magnesium, Phosphorus, and Potassium are critically low, and the blood glucose is elevated. What syndrome may this patient be at risk of?

Item Weight: 1.0

Question #: 3

You receive a consult for a patient in the ICU for a tube feeding. They are intubated and receiving mechanical ventilation after a motor vehicle accident. They are in critical condition, and have an advance directive that states they wish for artificial fluids and hydration. What feeding route do you recommend as your intervention?

Item Weight: 1.0

Question #: 4

You have assessed a patient in the hospital setting that has not been eating well. The patient's food and nutrition related history reveals an intake of <25% of their estimated nutrient requirements for about a month prior to hospitalization. You determine that this patient is a candidate for nutrition support. Which type of nutrition support will you recommend as a first intervention?

Item Weight: 1.0

Question #: 5

You have a patient that has hyperglycemia. They have no past medical history of Diabetes, but they have recently been hospitalized for pneumonia. They are intubated, and you are consulted to start a tube feeding. What type of tube feeding do you recommend?

Item Weight: 1.0

Question #: 6

While a patient is receiving speech therapy, oral foods are provided during daytime hours. To meet the patient's nutritional requirements, enteral nutrition via tube feeding is required during the night at a rate of 75 ml/hr over 10 hours. The night feeding is an example of:

Item Weight: 1.0

Question #: 7

A patient is receiving a tube feeding for the past six months. Over the course of the time the patient has been on the feeding, the feeding tube has clogged 4 times. The feeding tube is also used to provide the patient's medications. What intervention do you recommend?

Item Weight: 1.0
