# Supplementary material for: Using a virtual flipped classroom model to promote critical thinking in online graduate courses in the United States: a case presentation
Source: J Educ Eval Health Prof. 2022 Feb 28;19:5. doi: 10.3352/jeehp.2022.19.5 (PMC9008223; doi:10.3352/jeehp.2022.19.5)
Supplement: Supplementary file 2 — Supplement 2. A 5-question ungraded pre-quiz before and a 7-question graded post-quiz after class in an online pharmacology seminar course between September 2021 and December 2021 at the School of Health Professions, Rutgers The State University of New Jersey. [file jeehp-19-05-suppl2.pdf]

NUTR 5362 Pharmacology Seminar Module 9 Pre-Quiz

# of Questions: 5
Total Exam Points: 5.00

Question #: 1

Mr. Gray take phenytoin to manage a seizure disorder. Name two nutrition-focused physical findings might indicate phenytoin toxicity in Mr. Gray?

Item Weight: 1.0

Question #: 2

Anti-depressant medications may be prescribed for treatment/management of conditions beyond depression. Name two examples of when an anti-depressant might be used for treatment/management of a non-depression condition?

Item Weight: 1.0

Question #: 3

Remmie is a 10 year old boy with a seizure disorder. His BMI-for-age percentile is 15th%. The RDN and physician would like to see him gain some weight to be closer to the 25th%. Which medication could his physician prescribe to treat both the seizure disorder and support weight management goals?

Item Weight: 1.0

Question #: 4

Ms. Marigold was recently diagnosed with depression. Part of her depression is caused by her obesity (BMI 55 kg/m2). She recently enrolled in a high intensity lifestyle intervention program for weight management as she identified that her weight status has to do with poor diet, exercise, and lifestyle choices over the last 10 years. Which medication could her physician prescribe to treat both her depression and support her weight loss goals?

Item Weight: 1.0

Question #: 5

Ms. Zelma was recently diagnosed with bipolar disorder. Her physician plans to prescribe an anti-psychotic drug. Which metabolic health conditions that Ms. Zelma either has or might be at risk for developing must the physician consider when selecting an anti-psychotic drug for Ms. Zelma?

Item Weight: 1.0

NUTR 5362 Pharmacology Seminar Module 9 Post-Quiz

# of Questions: 7
Total Exam Points: 7.00

Question #: 1

Mr. Gray take phenytoin to manage a seizure disorder. Name two nutrition-focused physical findings might indicate phenytoin toxicity in Mr. Gray?

Item Weight: 1.0

Question #: 2

Anti-depressant medications may be prescribed for treatment/management of conditions beyond depression. Name two examples of when an anti-depressant might be used for treatment/management of a non-depression condition?

Item Weight: 1.0

Question #: 3

Remmie is a 10 year old boy with a seizure disorder. His BMI-for-age percentile is 15th%. The RDN and physician would like to see him gain some weight to be closer to the 25th%. Which medication could his physician prescribe to treat both the seizure disorder and support weight management goals?

Item Weight: 1.0

Question #: 4

Ms. Marigold was recently diagnosed with depression. Part of her depression is caused by her obesity (BMI 55 kg/m2). She recently enrolled in a high intensity lifestyle intervention program for weight management as she identified that her weight status has to do with poor diet, exercise, and lifestyle choices over the last 10 years. Which medication could her physician prescribe to treat both her depression and support her weight loss goals?

Item Weight: 1.0

Question #: 5

Ms. Zelma was recently diagnosed with bipolar disorder. Her physician plans to prescribe an anti-psychotic drug. Which metabolic health conditions that Ms. Zelma either has or might be at risk for developing must the physician consider when selecting an anti-psychotic drug for Ms. Zelma?

Item Weight: 1.0

Question #: 6

Mr. Levine is taking Nardil (phenelzine) a MAOI for treatment of depression. Which dietary recommendation should the RDN provide to this patient?

Item Weight: 1.0

Question #: 7

Ms. Patrick is taking lithium for treatment of bipolar depression. Her blood pressure is well-controlled and classified as normal. Which dietary recommendation should the RDN provide to this patient?

Item Weight: 1.0
